# Measured parental height in Turner syndrome—a valuable but underused diagnostic tool

**DOI:** 10.1007/s00431-017-3045-2

**Published:** 2017-12-18

**Authors:** Yasmine Ouarezki, Filiz Mine Cizmecioglu, Chourouk Mansour, Jeremy Huw Jones, Emma Jane Gault, Avril Mason, Malcolm D. C. Donaldson

**Affiliations:** 1 Etablissement Public Hospitalier Hassen-Badi, El-Harrach, Algiers, Algeria; 2 Paediatric Endocrinology and Diabetes Department, Kocaeli University, İzmit, Turkey; 3 University Hospital Abderrahim Harouchi, Casablanca, Morocco; 4 NHS Greater Glasgow and Clyde, Royal Hospital for Children, Queen Elizabeth University Hospital, Govan Road, Glasgow, G51 4TF UK; 5 College of Medical, Veterinary & Life Sciences, University of Glasgow, Glasgow, G12 8QQ UK; 6 Section of Child Health, Glasgow University School of Medicine, Glasgow, G12 8QQ UK; 7 Child Health Section of University of Glasgow School of Medicine, Queen Elizabeth University Hospital, Govan Road, Glasgow, G51 4TF UK

**Keywords:** Lower end of parental target range, Mid-parental height, Parental height, Sensitivity, Specificity, Turner syndrome

## Abstract

Early diagnosis of Turner syndrome (TS) is necessary to facilitate appropriate management, including growth promotion. Not all girls with TS have overt short stature, and comparison with parental height (Ht) is needed for appropriate evaluation. We examined both the prevalence and diagnostic sensitivity of measured parental Ht in a dedicated TS clinic between 1989 and 2013. Lower end of parental target range (LTR) was calculated as mid-parental Ht (correction factor 12.5 cm minus 8.5 cm) and converted to standard deviation scores (SDS) using UK 1990 data, then compared with patient Ht SDS at first accurate measurement aged > 1 year. Information was available in 172 girls of whom 142 (82.6%) were short at first measurement. However, both parents had been measured in only 94 girls (54.6%). In 92 of these girls age at measurement was 6.93 ± 3.9 years, Ht SDS vs LTR SDS − 2.63 ± 0.94 vs − 1.77 ± 0.81 (*p* < 0.001), Ht SDS < LTR in 78/92 (85%). Eleven of the remaining 14 girls were < 5 years, while karyotype was 45,X/46,XX in 2 and 45,X/47,XXX in 3.

*Conclusion*: This study confirms the sensitivity of evaluating height status against parental height but shows that the latter is not being consistently measured.

**Table:** 

**What is Known:** • *Girls with Turner syndrome are short in relation to parental heights, with untreated final height approximately 20 cm below female population mean*. • *Measured parental height is more accurate than reported height*.
**What is New:** • *In a dedicated Turner clinic, there was 85% sensitivity when comparing patient height standard deviation score at first accurate measurement beyond 1 year of age with the lower end of the parental target range standard deviation*. • *However, measured height in both parents had been recorded in only 54.6% of the Turner girls attending the clinic. This indicates the need to improve the quality of growth assessment in tertiary care.*

## Introduction

Short stature, defined as height below the 2.5th centile or more than two standard deviation scores (SDS) below the mean for a given population, is the commonest cause of referral to the endocrine clinic. The physician’s challenge is to accurately diagnose the relatively small proportion of short children with pathology by the most economic means possible, while sparing unnecessary investigation in the majority of naturally short children.

An important cause of short stature in girls is Turner syndrome, defined as loss of one sex chromosome or abnormality of the second X chromosome in at least one major cell line in a phenotypic female [3]. Short stature is a cardinal feature of Turner syndrome, untreated girls reaching final heights some 20 cm below the mean female height for the population concerned [11]. Early diagnosis is desirable partly because the short stature of Turner syndrome is amenable to treatment with growth-promoting therapy [15] but also because of the important implications for pubertal development and future fertility. Furthermore, late cardiovascular complications such as aortic dissection may be prevented when associated cardiovascular disorders such as aortic coarctation, bicuspid valve, and hypertension are identified [18].

Diagnosis of Turner syndrome is straightforward if the child presents with obvious dysmorphic features at birth such as pedal oedema and neck webbing with or without cardiac anomaly. However, girls presenting in childhood with short stature may have only subtle phenotypic traits which can be easily overlooked. It has been recommended that any girl who presents with short stature and/or premature ovarian failure should have a karyotype performed, in order to detect Turner syndrome [9]. While it is essential to request karyotype in any girl with ovarian failure, the feasibility and cost effectiveness of performing karyotype in all girls with short stature is open to question. This is because stature screening is neither specific nor wholly sensitive for Turner syndrome. In Scotland, for example, there were 53,802 live births for the year ending 31 March 2015 (http://www.nrscotland.gov.uk/statistics-and-data/statistics/statistics-by-theme/vital-events/general-publications/births-deaths-and-other-vital-events-quarterly-figures/1st-quarter-2015) so that an estimated 800 of the girls would be below the 3rd centile. Assuming a prevalence of 1 in 2000 live female births for Turner syndrome [8] it follows that only about 13 of the 800 short girls would have the condition, giving a very low specificity (1.6%) for crude short stature screening. Furthermore, childhood height in Turner syndrome is influenced by parental height [6] so that girls with tall parents may not be frankly short, especially during the early years.

Saari and colleagues have highlighted the value of combining three criteria—growth rate, height for age, and height in relation to parental height, in order to facilitate diagnosis of Turner syndrome [12]. We have shown previously that reported parental height is unreliable and that measured parental heights should be sought wherever possible [2].

Given the potential value of parental height in growth assessment, we wished to evaluate the quality of our growth service by determining the number and percentage of patients attending a dedicated Turner clinic in which height measurement had been carried out in both parents as well as the frequency of either only one or neither parent being measured. We have also examined the sensitivity of measured parental height in the diagnosis of Turner syndrome by comparing the height (Ht) SDS at the time of first accurate measurement with the Ht SDS of the lower end of the parental target range. Given that most girls with Turner syndrome show a degree of intrauterine growth restriction [5], a secondary aim of this study was to examine the sensitivity of the birth weight (BW) SDS in relation to specified cut-offs.

## Patients and methods

### Patients

The case notes of all girls with Turner syndrome, proven on karyotype, who had attended the Endocrine department at the Royal Hospital for Sick Children in Glasgow between 1989 and 2013 were reviewed and data collected up to and including 31 December 2014. The first height of each girl, after the first year of life, which had been recorded by accurate measurement using a stadiometer, and before any treatment with growth hormone had been started, was noted. The height of each girl’s mother and father was recorded as either measured, reported or not known/not recorded. The karyotype of each girl, analysed by cytogenetic analysis of lymphocyte culture counting 50 cells, was noted. Additional information gathered included birth weight and gestation, and associated disorders including cardiac malformations, gastrointestinal disorders such as inflammatory bowel and coeliac disease, middle and inner ear, endocrine, orthopaedic, and skin problems. Detail on learning difficulties in the patient population was considered to be beyond the scope of the study.

### Analysis of growth data

Mid-parental height (MPH) and parental target range were calculated as previously described [10], modified from Tanner’s system [16]. MPH was calculated as (mothers’ height + fathers’ height − 12.5 cm) ÷ 2. Lower end of parental target range (LTR) was determined by taking 8.5 cm as two standard deviations below MPH, i.e. MPH - 8.5 cm.

SDS values for the heights of the girls and parents were calculated using UK 1990 growth data [4]. MPH and LTR SDS were calculated for an adult age of 20 years, employing the LMS software developed by Professor Tim Cole using UK 1990 data http://www.healthforallchildren.com/?product_cat=software) [4]. BW SDS was calculated according to gestation using the LMS software.

The number of girls with Ht SDS < − 2 was recorded, to provide a measure of the sensitivity of short stature screening in the diagnosis of Turner syndrome.

When both parents had been measured, girls’ Ht SDS was compared with both LTR SDS and population height of − 2 SDS, in order to determine the prevalence of shortness relative to parental heights and the general population, and hence the sensitivity of family-specific shortness and short stature screening.

This exercise of comparing girls’ Ht SDS with LTR SDS was repeated for girls with available accurate first measurement in whom either one measured or one reported parental height; or both reported parental heights were documented.

The relationship between birth weight SDS, height at first measurement after the age of 1 year, and the LTR was also examined, taking BW SDS values < − 2 SDS, − 2 to < − 1.5 SDS and − 1.5 to < − 1.0 SDS, and examining the sensitivity of these birthweight cut-offs in the detection of Turner syndrome.

The data were analysed using Minitab statistical software (Version 13.1). Normality of distribution was tested using the Anderson-Darling method. If data were normally distributed, ANOVA was used for analysis and if non-parametrically distributed then Mann-Witney or Kruskall-Wallis and Mann-Witney test were used. Confidence level was 95% and *p* values were significant at < 0.05.

### Ethical aspects

The data were held electronically on a password-protected computer in the section of Child Health at the Royal Hospital for Sick Children in Glasgow. The study was registered with the Clinical Governance Support Unit of NHS (National Health Service) Greater Glasgow and Clyde as a quality improvement project.

## Results

Between 1989 and 2013, a total of 176 patients with a diagnosis of Turner syndrome, born between 1960 and 2013, were seen at the Royal Hospital for Sick Children in Glasgow. Four of the 176 girls were excluded because of insufficient information, which included no available karyotype in three. Diagnosis was by amniocentesis in 10 (5.8%), during the newborn period and later infancy in 62 (36%), from 1 to < 5 years in 15 (8.7%), between 5 and 10 years in 33 (19.1%), from 10 to 15 years in 43 (25%), 15–20 years in 6 (3.4%), > 20 years in one and unknown in two. Three patients had died during the study period (two cardiac deaths and one malignancy). Fifty cardiac anomalies were present in 33 (19.2%) patients comprising bicuspid aortic valve (22), aortic coarctation (14), aortic valve stenosis (5), anomalous venous drainage (4), atrial septal defect (2), aberrant systemic veins (2) and hypoplastic left heart (1).

Renal anomalies were identified in 22 (12.8%) including 13 horseshoe kidneys. A history of middle ear disease was documented in 94 (54.6%) while immune diseases included hypothyroidism in 11 (6.4%), type 1 diabetes in only one girl, inflammatory bowel disease in 3 (1.7%), coeliac disease in 3 (1.7%), scalp psoriasis in 6 (3.4%) and eczema in 5 (2.9%).

### Patient and parental height data (see Table 1)

Information on the 172 girls according to parental height measurement (both, one or neither) is shown in Table 1. Accurate height measurement at diagnosis and beyond the first year of life was available in 150 of these girls. Measured heights for both parents were found for 94 girls (see Table 1). One of these girls was excluded from analysis because of an unusually short height (− 5.58 SDS) recorded at the late age of 18.6 years and another because no height was available beyond the first year, leaving 92 girls. In 37 girls, only one parent had been measured, attributable to no contact with the father (8); parental illness (1); parent deceased (1); father never at clinic (2) and no obvious reason (25). No measured height was available from either parent in 41 girls, attributable to the child being fostered/adopted (3); no contact with parents (3); neither parent at initial clinic visit and not measured subsequently (3) and no obvious reason (32).

**Table 1:** Demographic and descriptive information on 172 patients with TS seen at the Royal Hospital for Sick Children in Glasgow between 1989 and 2013 in which data were collected up to 31 December 2014. Current age of girls in whom neither parent had been measured was significantly older than for girls in whom either one or both parents had been measured

	All girls [*n* 172]	Both parents measured [*n* 94]	Only one parent measured [*n* 37]	Neither parent measured [*n* 41]
Age on 1.1.15 (years)				
Mean ± SD	27.27 ± 9.97	24.93 ± 9.12*	25.77 ± 8.11**	34.01 ± 10.53*
Median (range)	27.94 (4.34–55.24)	25.90 (4.73–44.02)	26.82 (9.99–39.61)	35.10 (4.34–55.24)
Age at initial height measurement (years)				
Mean ± SD	7.68 ± 4.64	7.05 ± 4.05	10.26 ± 5.85	8.53 ± 6.55
Median (range)	7.69 (1.31–29.68)	7.2 (1.31–18.5)	11.39 (2.67–15.60)	6.65 (1.39–29.68)
Height SDS at initial measurement				
Mean ± SD	−2.70 ± 1.07 (n 146)	− 2.63 ± 0.94 (*n* 92^§^)	− 2.67 ± 1.26 (n 36)	− 2.92 ± 0.90 (n 19)
Median (range) [*n* = 172]	− 2.69 (−6.30^†^ to +1.79)	− 2.62 (− 4.71 to − 0.32)	− 2.71 (1.79 to − 6.30^†^)	− 2.95 (− 1.30 to − 4.88)
Birthweight (grams)	2805 (690–4060)	2800 (690–4060)	3010 (1540–3860)	2800 (1660–3660)
Median (range) [*n*]	[*n* 136]	[*n* 78]	[*n* 31]	[*n* 27]
Gestation (weeks)	40 (27–44)	39 (27–42)	40 (33–44)	40 (32–41)
Median (range) [*n*]	[*n* 139]	[*n* 79]	[*n* 32]	[*n* 28]
Karyotype [*n*]	[*n* 172]	[*n* 94]	[*n* 37]	[*n* 41]
45,X	69	34	16	19
45,X/46XiXq	29	17	5	7
45,X/46,XY	9	6	3	0
45,X/46,XX	9	5	3	1
45,X/47,XXX	11	7	4	0
45,X/46,XrX	14	7	3	4
46,XiXq	6	5	1	0
Other	23	13	2	8
Tested elsewhere	2	0	0	2

*p = 0.0001, **p = < 0.001

^†^Denotes girl with spina bifida and Turner syndrome in whom Ht SDS was − 6.30

^§^Denotes 2 girls excluded from height analysis owing to unusually short height (− 5.58 SDS) aged 18.6 years in one; no height available beyond the first year in the other

Girls in whom both parents had been measured tended to have been born more recently, diagnosed at a younger age and to be less short than girls in whom only one or neither parent had been measured. However, the only statistically significant differences found were in current age of the girls with measured parents (see Table 1).

### Sensitivity of Ht SDS < 2 SDS in girls with Turner syndrome (whole cohort)

Thirty (17.4%) of the 172 girls were not actually short (Ht SDS < − 2) at first measurement, giving a sensitivity of 82.6% for crude short stature screening. Median (range) Ht SDS in the 30 girls with Ht SDS ≥ − 2 was − 1.47 (− 2.00 to + 1.79). These girls were significantly younger at first measurement, median age 3.01 years compared with 8.56 years in the girls with Ht SDS < − 2 SDS (*p* = 0.01).

### Sensitivity of birthweight SDS < − 2, − 1.5 and − 1 in girls with Turner syndrome (whole cohort)

Data on birthweight were available in 136 of the 172 girls while gestation was available in 139. Median birthweight SDS was below average at − 0.91. After excluding one preterm girl with generalised oedema at birth who weighed 3400 g at 34 weeks’ gestation (BW SDS + 3.72), the median (range) birthweight for all girls was 2805 (690 to 4060 g (between − 3.6 and + 2.27 SDS). Fifteen girls had birthweight below − 2 SDS, 36 below − 1.5 SDS and 65 below − 1 SDS, giving sensitivities of 11, 26.7 and 48% for these cut-offs. Thirty-seven (27.4%) girls had birthweight between − 1 and 0 SDS, 24 (17.8%) between 0 and + 1 SDS; and only 9 (6.7%) above 1 SDS.

### Growth data on 92 girls in whom initial accurate height measurement after the age of 1 year and measured parental heights were available (Fig. 1, Tables 2 and 3)

**Fig. 1:**
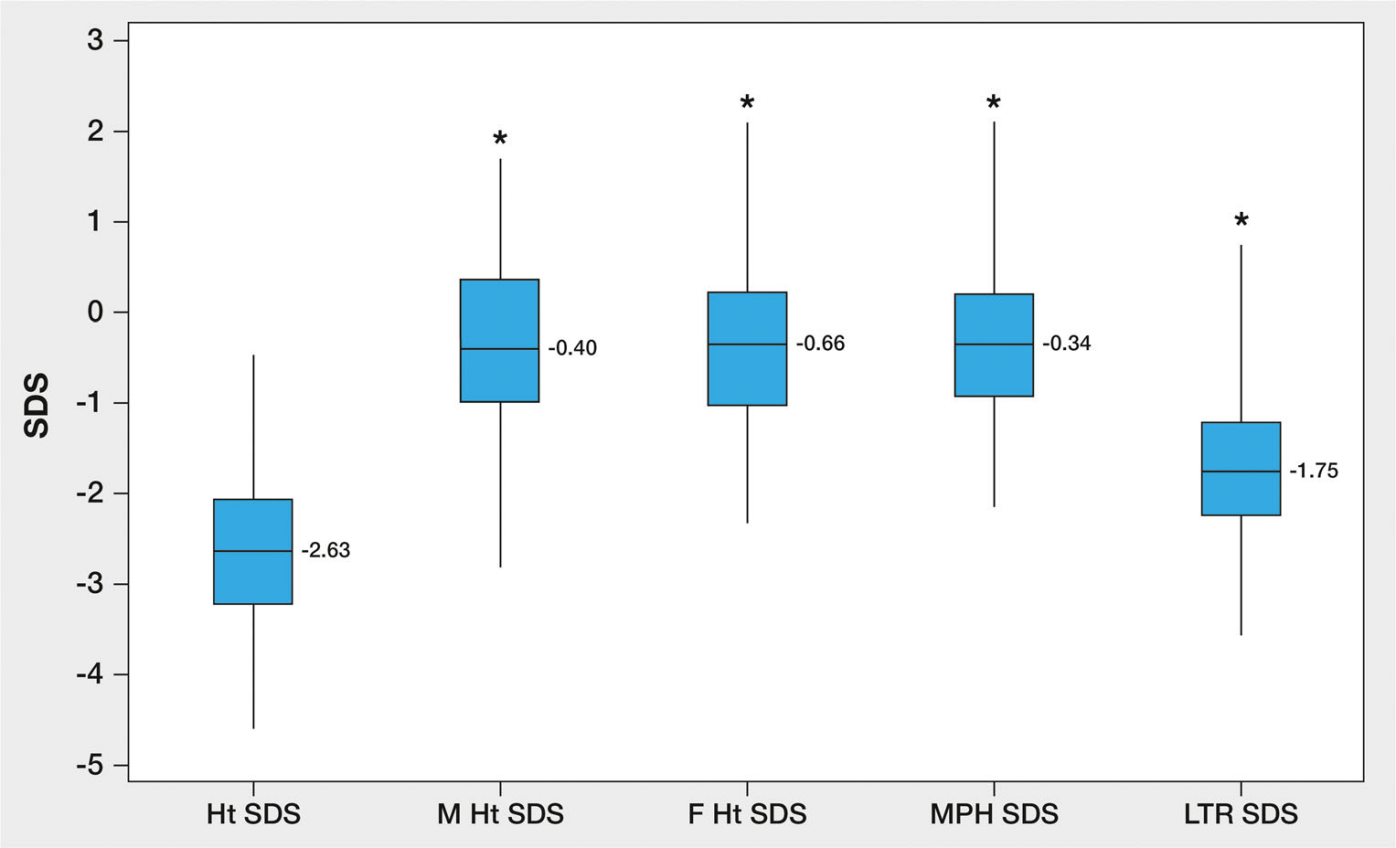
Box and whisker plot of height standard deviation score (Ht SDS) for first accurate measurement in 92 girls with Turner syndrome (P), and the Ht SDS for their mothers (M), fathers (F), mid-parental height (MPH) and lower end of target range (LTR). *p = < 0.001 using one-way ANOVA

**Fig. 2:**
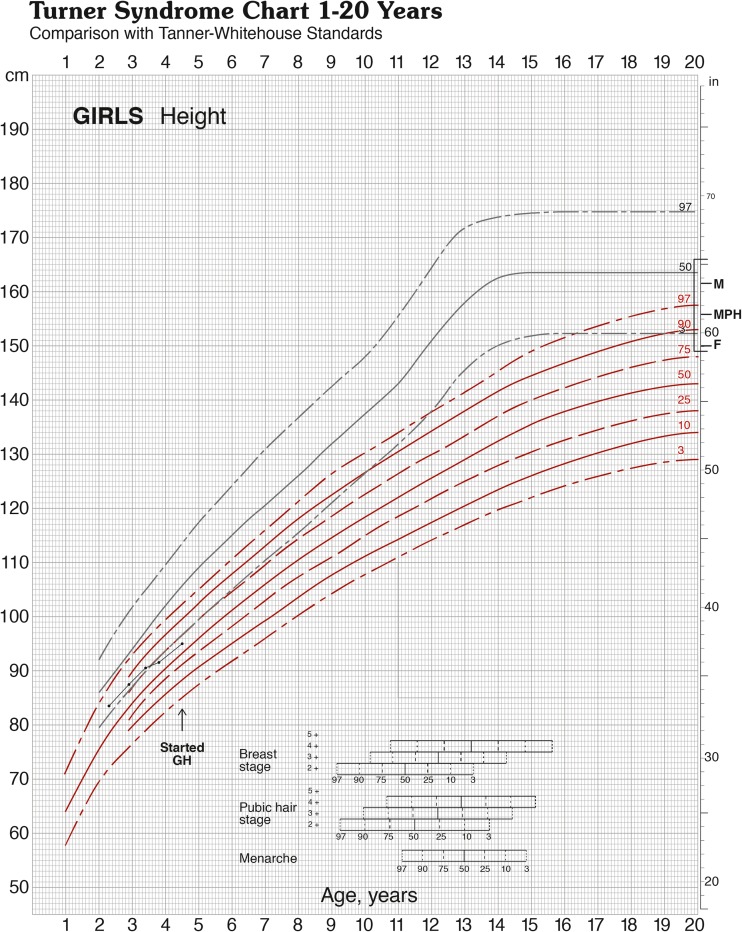
Height chart for Turner syndrome showing the growth pattern of a girl (asterisk in Table 3) in whom height fell within the mid-parental target range and general population at first accurate measurement. She then demonstrated decreased height velocity leading to short stature by 3.8 years and growth hormone treatment was started thereafter. (Figure is reproduced with kind permission from Castlemead Publications, Hertford, UK)

Mean± SD/median (range) age at first accurate measurement was 7.68 ± 4.64/1.31–29.7 years. All height data were normally distributed. As shown in Fig. 2, mean initial height SDS in the patients was significantly lower than maternal, paternal, and MPH SDS (*p* < 0.001, one-way ANOVA). Mean ± SD patients’ height SDS was also significantly lower than the LTR SDS: − 2.63 ± 0.94 vs − 1.77 ± 0.81 (*p* < 0.001, one-way ANOVA).

**Table 2:** Height standard deviation score (Ht SDS) of 92 girls with Turner syndrome in whom both parental heights were measured, grouped according to age at first measurement and to whether height standard deviation score (Ht SDS) was < or ≥ the lower end of parental target range SDS (LTR SDS)

Age at first accurate height measurement				
	1–5 years (*n* = 38)	5.1–10 years (*n* = 30)	10.1–16 years (*n* = 24)	1–16 years (*n* = 92)
< LTR SDS				
(*n*)	28	27	21	76
(%)	74	90	88	83
≥ LTR SDS				
(*n*)	10	3	3	16
(%)	26	10	13	17
Ht SDS < − 2				
(*n*)	27	25	22	74
(%)	71	83	92	80
Ht SDS ≥ − 2				
(*n*)	11	5	2	18
(%)	29	17	8	20

**Table 3:** Karyotype, height status and birthweight (BW) data in 14 girls with Turner syndrome whose initial accurate height measurement > 1 year of age fell above the lower end of the parental target range (LTR) height SDS

Karyotype	Age at first accurate measurement (years)	Ht SDS	LTR SDS	BW SDS	Comment
45,X	1.78	− 1.52	− 2.45	− 0.02	Father short (Ht SDS − 2.09)
45,X	2.40	− 1.42	− 2.71	N/A	
45,X	2.46	− 0.62	− 1.25	0.09	
45,X/47,XXX	2.51	− 0.60	− 1.88	N/A	
45,X	2.69	− 0.57	− 1.43	N/A	
45,X/47,XXX	3.01	− 0.75	− 0.83	− 2.74	
45,X	3.23	− 1.25	− 1.31	1.08	
45,X	3.27	− 2.15	− 2.78	1.98	Father short (Ht SDS − 2.2)
45,X/47,XXX	4.24	− 1.19	− 1.73	− 1.76	
45,X	4.93	− 2.15	− 2.66	− 1.30	
45,X/46,XX	4.99	− 0.32	− 1.72	N/A	
45,X/46,Xr(X)	7.20	− 1.05	− 1.46	N/A	
45,X/46,XX	9.08	− 2.75	− 2.85	N/A	
45,X	9.10	− 2.02	− 2.16	− 1.34	

Data are ranked according to age at initial height measurement

*N/A* not available

In addition to the 92 girls in whom accurate height and measured parental height in both parents were available, accurate initial height was available in 31 girls with only one parent measured (the mother in all but one case) and 15 girls in whom both parental heights were reported. The difference between LTR and Ht SDS for the 92 girls with both parents measured was (− 1.77) − (− 2.67) = 0.9; for the 31 girls in whom only one parent was measured the difference was (− 1.92) − (− 2.47) = 0.56; and for the 15 girls in whom both parental heights were reported the difference was (− 1.91) − (− 2.83) = 0.92. None of these differences achieved statistical significance.

Table 2 shows that in the 92 girls where measured height was available for both parents, Ht SDS at first accurate measurement was below LTR SDS in 78 (sensitivity 85%) while 14 (15%) girls had Ht SDS ≥ LTR SDS. Eleven of 38 girls aged < 5 years had Ht SDS ≥ LTR SDS (71% sensitivity). Median (range) age at first measurement in these 11 girls was 3.0 (1.8–4.99) years, five of the girls being under 3 years of age. Only three girls in the 5–10 year range had Ht SDS > LTR SDS (90.3% sensitivity) and none aged > 10 years (100% sensitivity).

Table 3 gives details on the 14 girls with Ht SDS > LTR SDS. More than half of the 14 girls had 45,X monosomy (*n* = 8). Five had karyotypes known to be associated with a milder phenotype so that 2 of the 5 girls with 45,X/46,XX and 3 of the 7 girls with 45,X/47,XXX had initial Ht SDS > LTR SDS. Only one of these five girls was actually short, i.e. < − 2SD.

## Discussion

We have examined a group of girls with Turner syndrome from the West of Scotland in order to audit the frequency with which parental height had been measured and also to test the sensitivity of height at first measurement in the girls in relation to their measured parental heights.

In this unselected cohort, the prevalence of cardiac and renal disease is lower, albeit of similar order, while the 54.6% prevalence of middle ear disease is similar to that previously reported [13], indicating that our patients are reasonably representative of Turner syndrome. An unexpected finding in the course of this study is a relatively high prevalence of scalp psoriasis (3.4%), an observation which merits further study.

Both parents had been measured in 94 of the 172 girls—only just over half. Those with measured parental heights were not different from girls in whom only one or neither parent had been measured except in age—the girls with no measured parental height were significantly older. We infer that girls born earlier, e.g. in the 1970s and before, would be more likely not to have both parents measured. However, it was common, even in recently diagnosed patients, for one parent (usually the father) not to be measured. Parental height is usually measured at the initial visit to the clinic and if only one parent is present the other parent may not be measured subsequently, particularly when the diagnosis of Turner syndrome has already been made. However, measured parental height is not only of value in diagnosis but also for determining the target height for the girl with or without growth hormone treatment.

Analysis of the difference between parental LTR SDS and girls’ Ht SDS in girls with both parental heights measured, one parental height measured, and both parental heights reported is interesting with differences of 0.9, 0.56, and 0.92 for the three groups. We interpret this as showing that when the fathers’ report their height, they overestimate it, leading to a reduction in the LTR/Ht SDS difference (0.56 vs 0.9). We also speculate that when the heights of both parents are reported, the mothers tend to underestimate their height while the fathers tend to overestimate height as previously reported [2] so that the errors cancel one another out. Clearly, it is not desirable to subject girls with a growth disorder such as Turner’s syndrome to the vagaries of reported height.

The data from this study raise wider questions concerning the adequacy of clinical assessment in children with growth problems in general, and not just those with Turner syndrome. If only just over half the parents of Turner girls have been measured in a tertiary referral clinic, it is likely that this figure is similar for other disorders such as hypopituitarism and almost certainly lower in the general paediatric clinic. Yet, this study confirms the value of comparing patient height with parental height, while previous work has shown the superiority of measured rather than reported parental height [2]. A more rigorous approach is required to ensure that both parents are measured in clinic and that missing heights from the initial visit are secured at a later stage where possible.

Although there was no documented reason for non-measurement of one or both parents in 57 girls, a significant proportion of parental heights will be unavailable at the time of first measurement due to factors such as separation and divorce. In a separate study, we have evaluated the feasibility of obtaining parental heights at the time of birth in small babies as well as securing accurate length measurement [14]. We would also make the case for measuring not only mothers antenatally as is standard obstetric practice in the UK but also their partners, transcribing this information onto the neonatal growth record as part of good practice in the promotion of child health.

We should stress that in our study population, short stature was by no means the key factor in the diagnosis of Turner syndrome in every patient. On the contrary, the diagnosis had been made before 1 year in 40% of the girls, and would have been related to features such as dysmorphism, lymphoedema and to problems such as aortic coarctation rather than short stature. Thus, the present study is not concerned with diagnosis in Turner syndrome per se*.* Rather, it constitutes an exercise in determining how many of the girls in our clinic were and were not below their parental height target range at the first age of accurate measurement, and includes those who had been already been diagnosed through features other than short stature. On the other hand, some girls were diagnosed late with short stature and delayed puberty, the prevalence in our study group being of similar order to the study of Massa et al. [7] who reported diagnosis aged > 12 years in 54 (22%) of 242 girls compared with nearly 30% aged > 10 years in ours.

It should also be noted that even when the diagnosis of Turner syndrome has been made, parental height status is still relevant in setting a standard for height which the family can realistically expect and which the clinician should aim to achieve, i.e. the lower end of the target range.

This study confirms the limited sensitivity of both short stature screening and birthweight analysis in the diagnosis of Turner syndrome. The sensitivity of detecting girls who were < − 2 SDS in height at first measurement was 82.1%, indicating that screening girls who are actually short will miss nearly 20%. Moreover, although birthweight was below average in 102 of 136 girls (75%), only half had birthweight < − 1 SDS, and only 11% below − 2 SDS, these cut-offs being insufficiently sensitive or specific to be of practical use. Our findings are in keeping with the work of Hagman et al. [5] and show that although a degree of intrauterine growth restriction is common in Turner syndrome, this feature of the condition is not helpful in diagnosis. Fifteen girls had birthweight below − 2 SDS, 36 below − 1.5 SDS and 65 below − 1 SDS, giving sensitivities of 11, 26.7 and 48% for these cut-offs. Thirty-seven (27.4%) girls had birthweight between − 1 and 0 SDS, 24 (17.8%) between 0 and + 1 SDS; and only 9 (6.7%) above 1 SDS.

The sensitivity of measuring Ht SDS in 92 girls against parental heights was slightly greater than that obtained using the general population cut-off of < 2 SDS—83 vs 80% (Fig. 2)—and similar to the figure of 82.6% obtained for detecting short stature in the whole cohort. However, the former method is much more specific since it takes account of parental heights and is hence less likely to miss girls with tall parents. When the sensitivity of Ht SDS vs LTR SDS was examined in relation to age group it was found that 11 of 14 girls who were within the parental target range SDS were below the age of 5 years, with only 3 aged 5–10 years and none above 10 years (see Table 2). This finding is in keeping with the documented growth pattern shown by Turner-specific growth charts (see Fig. 2) which shows that growth velocity is typically low-normal for the first 3–4 years, with height falling away from the 3rd centile thereafter. Individuals with 45,X/46,XX genotype are known to be more mildly affected [17] as are those with the 45,X/47,XXX genotype [1] so that these girls will tend to be within the upper half of the Turner-specific charts and indeed may remain within the normal population range. We advise monitoring growth in girls with Turner syndrome using both condition-specific and population-based growth charts. Thus, while our study confirms the integral place of parental height measurement in the assessment of girls with Turner syndrome, it is evident that this aspect of auxology should not be over-relied on, particularly in the young patient. Also, Wright and Cheetham have pointed out the limitations of the mid-parental height model, especially when parents are particularly tall or short, although their study relied on reported rather than measured parental height [19]. The criteria described by Saari et al. have the advantage of taking height velocity as well as height status and parental heights into account [12]. However this model is not readily applicable to the initial presentation unless accurate previous measurements are available and may not always be practical in a busy clinic.

In conclusion, parental height measurement is a sensitive and valuable adjunct to growth assessment in children with growth problems, including but not confined to children with Turner syndrome. Applying the mid-parental height and target range to the child’s growth assessment is not a fail-safe system, particularly in preschool children, and should be regarded as only one aspect of good clinical practice. Finally, there is a clear need to measure parental height more rigorously in our clinic, and this is likely to be the case in other centres.

## Abbreviations

NB: Apart from in the abstract the term Turner syndrome is given in full
ANOVA: Analysis of variance
BW: Birth weight
Ht: Height
MPH: Mid-parental height
LTR: Lower end of parental target range
SDS: Standard deviation score
